# Investigating microRNA-Target Interaction-Supported Tissues in Human Cancer Tissues Based on miRNA and Target Gene Expression Profiling

**DOI:** 10.1371/journal.pone.0095697

**Published:** 2014-04-22

**Authors:** Wan J. Hsieh, Feng-Mao Lin, Hsien-Da Huang, Hsiuying Wang

**Affiliations:** 1 Institute of Statistics, National Chiao Tung University, Hsinchu, Taiwan; 2 Department of Biological Science and Technology, Institute of Bioinformatics and Systems Biology, National Chiao Tung University, Hsinchu, Taiwan; The Perinatal Institute, Cincinnati Children’s Hospital Medical Center and University of Cincinnati, United States of America

## Abstract

Recent studies have revealed that a small non-coding RNA, microRNA (miRNA) down-regulates its mRNA targets. This effect is regarded as an important role in various biological processes. Many studies have been devoted to predicting miRNA-target interactions. These studies indicate that the interactions may only be functional in some specific tissues, which depend on the characteristics of an miRNA. No systematic methods have been established in the literature to investigate the correlation between miRNA-target interactions and tissue specificity through microarray data. In this study, we propose a method to investigate miRNA-target interaction-supported tissues, which is based on experimentally validated miRNA-target interactions. The tissue specificity results by our method are in accordance with the experimental results in the literature.

**Availability and Implementation:**

Our analysis results are available at http://tsmti.mbc.nctu.edu.tw/ and http://www.stat.nctu.edu.tw/hwang/tsmti.html.

## Introduction

MicroRNA is a short non-coding RNA that is approximately 22 nt, which suppresses gene expressions via translational suppression or mRNA degradation by binding to 3′-untranslated regions (3′UTR). The discovery of the first miRNA from Caenorhabditis elegans in 1993 inspired a wide variety of miRNA studies [1]. At present, approximately 21,264 miRNAs have been discovered in many species.

To study the regulation between miRNAs and genes, miRNA target sites are usually predicted by miRNA target prediction tools. Many computational target prediction tools, such as MiRanda [2],TargetScanS [3]–[5] and RNAhybrid [6], have been developed. In addition, several statistical methods have also been applied to build a network of associations between miRNAs and their target mRNAs [7]–[10]. Usually, miRNA target prediction tools predict many potential target sites. To reduce the number of false-positive target sites, predicted miRNA target sites should be confirmed by experiments. Generally, miRNA-target interactions (MTIs) can be confirmed by reporter assays, Western blot, microarray experiments, pSILAC or qRT-PCR. Moreover, many databases, such as miRTarBase [11], TarBase [12], miRecords [13] and miR2Disease [14], have been designed for storing experimentally validated MTIs. In particular, the miRTarBase (version 2.1) database has collected approximately 3,500 manually curated experimentally validated MTIs, including 657 miRNAs and 2,297 target genes among 17 species from 985 research articles [11]. The TarBase 5.0 database has stored approximately 514 MTIs that were extracted from 203 papers [12]. The miRecord database, which includes 1,529 experimental interactions, is composed of experimentally validated miRNAs and predicted MTIs [13]. The miR2Disease database is aimed at storing experimentally validated MTIs, which are deregulated in various human diseases [14]. Among these databases, miRTarBase provides more updated MTIs than the other databases.

Importantly, miRNAs have been observed to be tissue-specific in many studies. For example, miR-122 can only be detected in liver tissues and is undetectable in all other tissues [15]; the expression of miR-122 in hepatocellular carcinoma is relatively lower than it in healthy liver [16]; miR-1 and miR-143 are preferentially expressed in heart and colon tissues, respectively [15], [17]; miR-126 is an endothelial-specific miRNA that regulates vascular integrity and angiogenesis [18]; miR-195 and miR-200c are specifically expressed in lung tissues [19]. In addition, some miRNAs are biomarkers for detecting cancers, such as miR-221, -100, -125b and -21 in pancreatic cancer [20].

Although many researchers have indicated that many miRNAs have tissue-specific expression [15]–[20], no systematic methods have been established in the literature to investigate the highly negative correlations between an miRNA and its target genes in a group of specific tissues through microarray data. Analyzing the correlation of expressions between miRNAs and mRNAs is one of the methods that has been applied for increasing the confidence of predicted miRNA target sites [21]–[28]. In this study, we have developed a statistical method to determine microRNA-target interaction-supported tissues (MTI-supported tissues) based on experimentally validated miRNA-target interactions. The MTI-supported tissues of an miRNA is a group of tissues that this miRNA and its targets express in these tissues.

The major aim of this study is to investigate the MTI-supported tissues that are based on experimentally validated miRNA-target interactions in the miRTarBase database. At http://tsmti.mbc.nctu.edu.tw, we briefly describe how the proposed method is applied to identify MTI-supported tissues. The major analytical results and the materials in this paper are presented on this website.

## Materials and Methods

**Figure 1:**
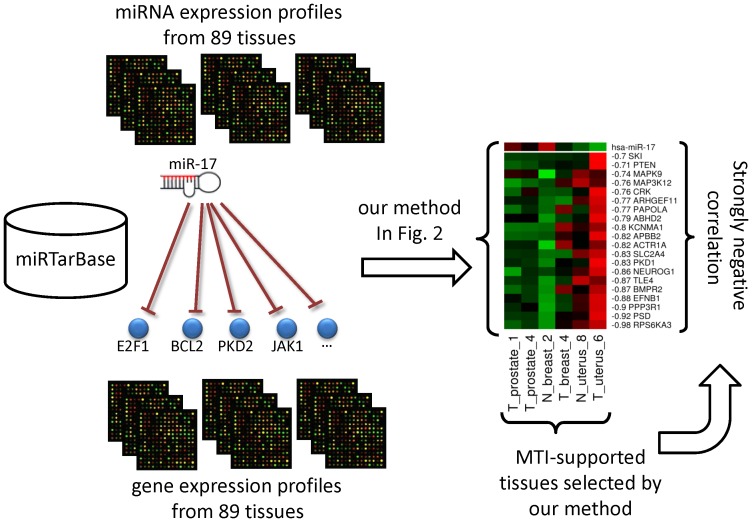
Tissue selection pipeline for finding MTI-supported tissues of miR-17 based on experimentally validated MTIs. Experimentally validated MTIs sharing the same miRNA were selected from the miRTarBase database. The correlations of MTIs across a combination of tissues, which were selected by our method, are strongly negative.

The conception of the proposed procedure is briefly shown in Figure 1. We use data sets [29] to illustrate our methods. This data set includes the miRNAs expression profiles and mRNA expression profiles for 89 samples of 11 organs from tumor or normal tissues. The samples of 11 organs are summarized in Table 1, including 68 tumor tissues and 21 normal tissues. The mRNA expression profiles that were published in 2005 consist of two microarray platforms, GPL80 and GPL98, which represent 16,063 genes across 89 tissues [29]. Because partial mRNA expression levels are missing, only 12,766 of these genes are used in our studies. The miRNA expression profiles (GSE2564) are composed of the expression data for 288 miRNAs across 249 tissues [29]. After eliminating duplicate and redundant data, we only use data for 163 miRNA across 89 tissues. With the data for an miRNA, we intend to investigate the MTI-supported tissues to determine whether the miRNA is functional across these tissues based on experimentally validated MTIs. As mentioned in the Introduction, the miRTarBase database is more informative than other databases. We apply the miRTarBase version 2.1 database [11] to obtain experimentally validated MTIs, which can be accessed at “http://mirtarbase.mbc.nctu.edu.tw/cache/download /2.1/miRTarBase_MTI.xls”. According to the miRNAs that were recorded in GSE2564, we select 743 experimentally validated MTIs and analyze the correlations between miRNA and mRNA expression profiles across different tissue sets.

**Table 1:** Tumor tissues and normal tissues.

Tissues	Tumor	Normal
Colon	7	4
Uterus	10	1
Prostate	6	6
Pancreatic	8	1
Ovary	5	0
Lung	5	2
Breast	6	3
Bladder	6	1
Mesothelioma	8	0
melanoma	3	0
Kidney	4	3
Total	68	21

Before analyzing the expression data, we first normalize the miRNA and mRNA expression data across 89 tissues. The data pre-processing method is provided in the Supplementary Data. Some data pre-processing results are presented in Figures S1 and S2 in File S1. After the data pre-processing, the top 23 miRNAs with a target number that is greater than or equal to 10 and with a number of tissues with expression levels that were greater than 7.25 are selected and are listed in Table 2. The expression level of 7.25 is the cutoff point that was used in Huang *et al.* (2007) [7]. In Table 2, four miRNAs, hsa-miR-122, hsa-miR-124, hsa-miR-155 and hsa-miR-133a, are ignored because there are not enough samples for these miRNAs that could be utilized for the analysis. Therefore, we analyze 19 miRNAs in this study.

**Table 2:** The list of miRNAs with equal to or more than 10 targets among 743 experimentally validated MTIs and the average correlations.

SN	miRNA	the number oftargets	the number oftissue with expressionlevel larger than 7.25	the number of selectedMTI-supported tissues	average correlationbetween an miRNAand its targets across all89 tissues (A)	average correlation betweenan miRNA and itstargetsacross selected MTI-supportedtissues (S)	S-A
1	hsa-miR-21	43	89	5	0.022	−0.378	−0.4
2	hsa-miR-122	34	2	–	–	–	–
3	hsa-miR-124	32	10	–	–	–	–
4	hsa-miR-1	29	42	5	−0.043	−0.462	−0.419
5	hsa-miR-17	24	67	6	0.04	−0.496	−0.536
6	hsa-miR-155	20	3	–	–	–	–
7	hsa-miR-145	18	87	5	0.019	−0.301	−0.32
8	hsa-miR-34a	16	75	5	−0.006	−0.524	−0.518
9	hsa-miR-125b	15	86	6	−0.047	−0.423	−0.376
10	hsa-miR-16	14	89	7	−0.015	−0.408	−0.393
11	hsa-miR-221	14	87	5	0.029	−0.346	−0.375
12	hsa-let-7a	14	89	8	−0.029	−0.437	−0.408
13	hsa-miR-29b	13	87	5	0.035	−0.343	−0.378
14	hsa-miR-210	13	54	5	0.049	−0.631	−0.68
15	hsa-miR-20a	12	80	6	−0.026	−0.547	−0.521
16	hsa-miR-106a	11	73	5	0.015	−0.555	−0.57
17	hsa-miR-29c	11	88	5	−0.072	−0.66	−0.588
18	hsa-miR-222	10	58	5	−0.032	−0.463	−0.431
19	hsa-miR-26a	10	87	5	−0.004	−0.568	−0.564
20	hsa-miR-24	10	88	5	−0.007	−0.577	−0.57
21	hsa-miR-15a	10	86	6	0.017	−0.566	−0.583
22	hsa-miR141	10	66	5	−0.019	−0.566	−0.547
23	hsa-miR-133a	10	17	–	–	–	–

“−” denotes there are no MTIs supported tissues selected in this study. The 23 miRNAs with the number of targets.

Before introducing the method, we first describe the motivation for proposing the method. Previous studies have shown that miRNAs down-regulate their targets [16], [30], which results in a negative correlation of the microarray expressions between an miRNA and its target interactions. However, Figure S1 in File S1 reveals that the absolute values of most correlations of the miRNAs and their target interactions across all 89 tissues are not significantly large. We, therefore, conclude that the down-regulation of an miRNA occurs in some tissues.

For an miRNA, we first find the associated interactions through the microarray dataset and the miRTarBase database and then calculate the correlations between the miRNAs and their targets. Thus, an miRNA is associated with a correlation set, which includes the correlations between this miRNA and its targets. Because there is more than one target interaction that is associated with an miRNA, our goal is to integrate several correlation coefficients between this miRNA and their targets to find MTI-supported tissues. Therefore, to determine the MTI-supported tissues of this miRNA, we propose using a criterion that is based on the two factors in the correlation sets: (i) the average correlation coefficient and (ii) the proportion of negative correlation coefficients. Due to the down-regulation of the miRNA to its target interaction, for a set of true MTI-supported tissues of an miRNA, we expect that the expression data between this miRNA and its target interactions would be highly negatively correlated. Therefore, we expect that the true MTI-supported tissues should satisfy the hypothesis that the average correlation coefficients are strongly negative and that the proportion of negative correlation coefficients is large.

To describe the proposed method, we first introduce some notations. We denote the 89 tissues as 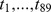 and the 19 miRNAs as 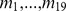. The expression value of an miRNA 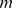 and an mRNA 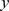 across the 89 tissues is denoted as 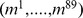 and 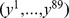. Let 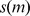 denote the target interaction (mRNA) number that corresponds to this miRNA from the miRTarBase database.

Let 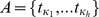, 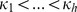, be a set of 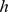 tissues of the 89 tissues, where 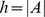 is the size of sample set A. The correlation coefficient of the miRNA *m* and the mRNA *y* across a sample set *A* is defined as
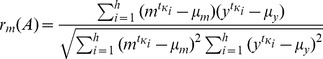
where 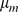 and 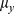 denote the means of 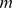 and *y* across the tissues in the set 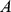, respectively.

Let 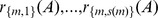 denote the correlation coefficients of this miRNA and its target interactions across the tissues in set 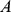, and let 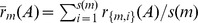 denote the average of these correlation coefficients across the tissue set 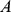. In addition, let 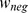 be the number of negative value of the 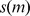 correlation coefficients. Then, we define the negative correlation coefficient proportion as.
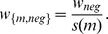



For the miRNA 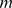, the goal of this study is to find a tissue set 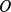 such that 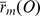 is strongly negative. In addition, because there are 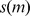 correlation coefficients for this mRNA, we must require that the proportion of negative correlation coefficients among these 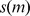 correlation coefficients is greater than a threshold. That is, we intend to find a tissue set such that 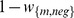 is small. Thus, we propose using the loss function.
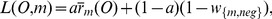
(1)to select a tissue set 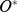, such that the minimum of the loss function occurs at 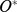, where *a* is a constant between 0 and 1, that is,



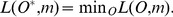
(2)In the loss function (1), *a* is used to adjust the weights of 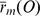 and 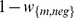. To find 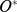 satisfying the condition (2), it is difficult to directly calculate the 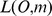 value for all sets of 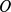. For a set 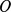 with 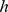 elements, there are 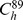 combinations. Because the range of the 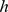 value is from 2 to 89, there is a total of
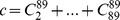
possible selections of a set 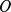. The calculation complexity is too high to obtain the true 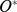. Because the total possible combination 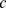 is too large, we may slightly relax the condition (2) to be
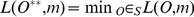
(3)where S is a set of *O*, which is restricted to have 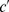 elements instead of 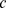 elements, where 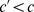. In the following analysis, 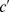 is selected to be 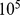. To select an adequate 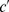 value, we had tested many different 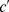 values and found that the selected tissue of using 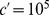 is almost the same with the selected tissues of using 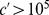 for many cases. Thus, we adopt 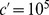 in this analysis.

When sampling tissue sets, a heuristic method or a brute-force method can be adopted. If highly confident MTI-supported tissues for an miRNA are available from the literature or other resources, we suggest directly choosing tissue sets including these tissues in implementing the algorithm, which can prune the search space. Otherwise, the brute-force sampling is suggested to avoid obtaining not objective results. Since the suggested sampling size is 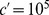, it is not very time-consuming in implementing the algorithm when we use the brute-force sampling method.

The steps of finding the 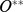 under condition (3) is presented in the following algorithm. Before preceding the algorithm, we must specify the constant 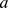 in condition (1).

To evaluate the performance of our proposed algorithm, we adopt a permutation test and a clustering analysis [31] to show the superiority of the proposed algorithm. Both methods are described in the Supplementary Data. Some comparison results are presented in Figure S3 in File S1 and Table S1 in File S1. The results of the two methods uphold our proposed algorithm as an efficient approach to select valid MTI-supported tissues of an miRNA.

### 2.1 Algorithms

#### Algorithm for tissue prediction: correlation loss function algorithm

Suppose that an miRNA *m* has 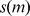 MTIs.

**Step1:** Randomly select a set *O* with 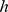 tissues.

**Step2:** Calculate the correlations between this miRNA and the 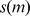 mRNAs across the tissues in *O*. Then, calculate the mean of these correlations, 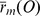 and the proportion of negative correlations, 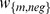.

**Step3:** Use the values 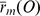 and 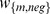 to calculate (1).

**Step4:** Repeat Steps 1–3 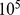 times. We obtain 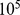 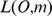 values.

**Step5:** Find out the minimum value of the 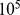 values, say 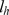. List the tissue set, say 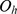, corresponding to this 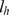 value, that is 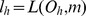.

**Step6:** Repeat Steps 1–5 for different 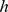 to obtain different 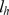 values. Find the minimum value of these 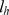
s, say 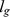. Then, the tissue set 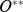 corresponding to this 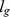 value is the MTI-supported tissues set.

In addition to only considering the correlations of the expressions between miRNAs and mRNAs, DNA copy-number and promoter methylation at the mRNA gene locus on mRNA expression may influence the miRNA-mRNA expression association. We have provided R-codes which include the other factors in the loss function. The readers can access the R codes at http://tsmti.mbc.nctu.edu.tw/lossfunction2.txt.

**Figure 2:**
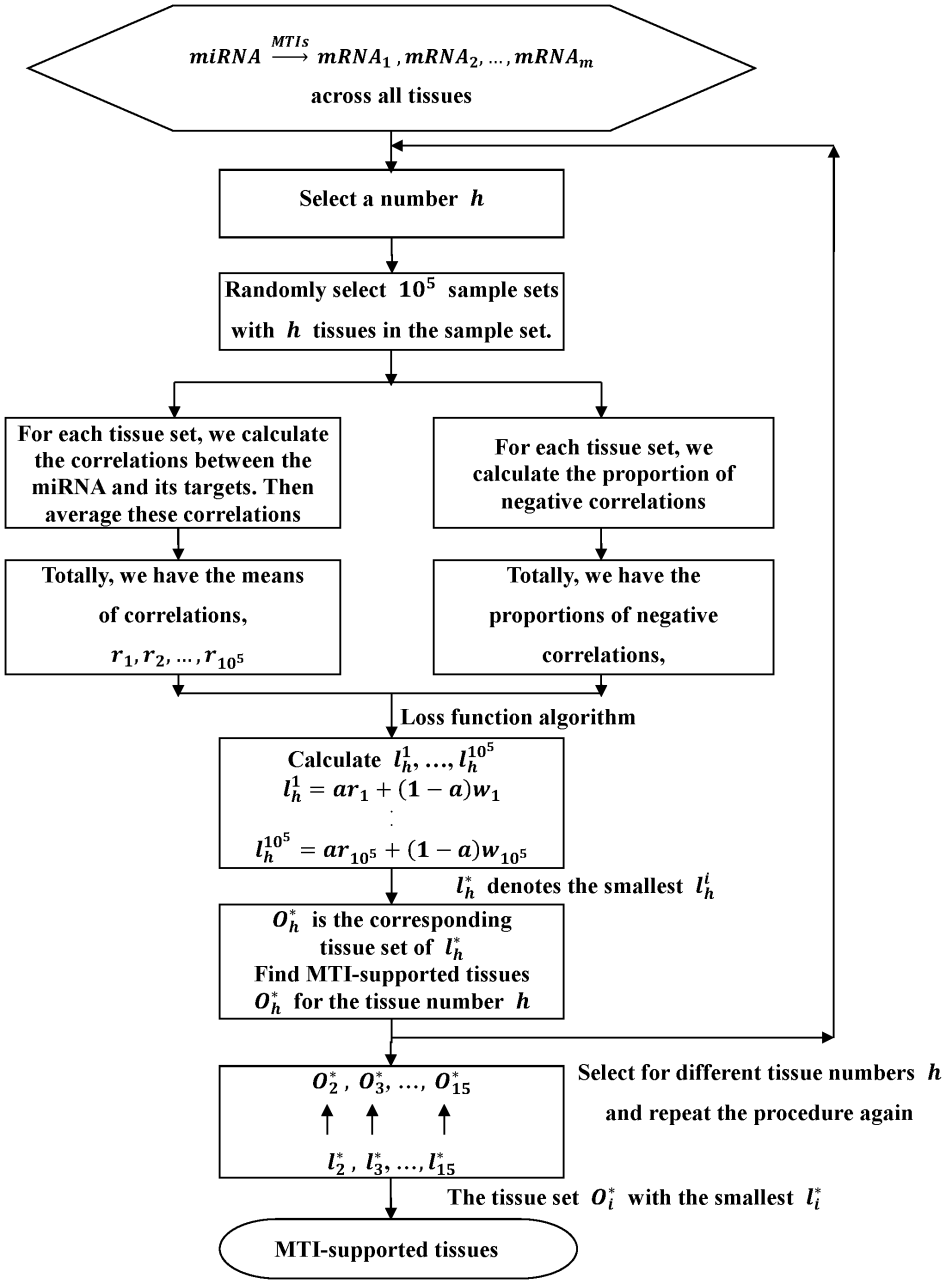
The flowchart of the proposed algorithm for finding MTI-supported tissues.

Because the algorithm is based on the loss function (1) to evaluate the performance of the correlation, we called this algorithm the correlation loss function algorithm. The steps of our algorithm are described in the flowchart in Figure 2.

Practically, for an miRNA and its targets, we are not sure how many tissues should be selected. First, we begin by selecting 3 tissues from all tissues but do not select one tissue because the correlation between an miRNA and its targets across one tissue cannot be calculated. To find the optimal tissue number of different miRNAs, we select the tissue number from 3 to 15.

**Figure 3:**
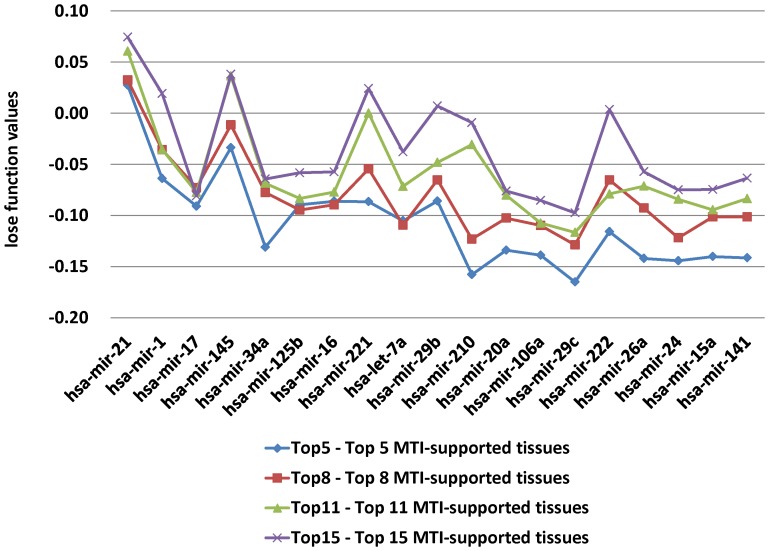
The loss of function values for different numbers of MTI-supported tissues.

In Figure 3, we illustrate the loss of function values with 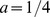 of each miRNA and its targets across 5 tissues, 8 tissues, 11 tissues and 15 tissues, respectively. From the calculation results, we find that the loss function value across more than 15 tissues is larger than the loss function value across less than 15 tissues. Therefore, we do not consider the case with tissue number that is greater than 15, and only chose 3 to 15 tissue numbers to find the optimal tissue number. The results indicate that the optimal tissue number that is derived by the algorithm depends on the miRNAs.

This paper mainly presents the results when 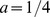. We have used other 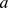 values such as 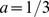 in the loss function to select tissues. The results for 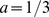 are similar to those for 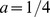. Because 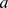 is used to adjust the weight between the mean of the correlations 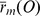 and the proportion of positive correlations 1-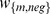, and we are more concern the proportion of positive correlations, we put more weight on the second term. In this study, the results using 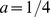 lead to good performance. Therefore, 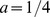 is a suggested value in using this algorithm. For other real applications of this algorithm, the readers can use training data to obtain a suitable 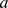 value.

In addition, to make a more objective analysis in selecting the MTI-supported tissues, instead of selecting a tissue set among tissue sets for 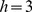 to 15 which minimizes the loss function (1), we proposed a method to rank tissues by the following two steps. The first step is to find the 13 tissue sets which minimize the loss function corresponding to 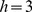 to 15, respectively. The second step is to rank the tissues appeared in these 13 tissue sets according to their occurrence numbers among the 13 tissue sets. The tissue with the most occurrence frequency is ranked first and so on. The ranking results are presented in Table 3, which can provide information on the significance of the MTI-supported tissues.

**Table 3:** The MTI-supported tissues from rank 1 to 8.

miRNA	hsa-mir-21	hsa-mir-1	hsa-mir-17	hsa-mir-145	hsa-mir-34a	hsa-mir-125b	hsa-mir-16	hsa-mir-221	hsa-let-7a	hsa-mir-29b	hsa-mir-210	hsa-mir-20a	hsa-mir-106a	hsa-mir-29c	hsa-mir-222	hsa-mir-26a	hsa-mir-24	hsa-mir-15a	hsa-mir-141
the number oftargets	5	5	6	5	5	6	7	5	8	5	5	6	5	5	5	5	5	6	5
1	T_kidney_1	N_prostate_6	N_uterus_8	T_uterus_1	T_breast_3	T_prostate_5	T_breast_5	T_ovary_6	T_bladder_1	T_ovary_5	N_colon_4	T_bladder_1	T_uterus_2	T_mesothelioma_4	T_colon_3	T_pancreas_5	T_melanoma_1	T_ovary_5	N_pancreas_1
2	T_ovary_3	T_mesothelioma_1	T_uterus_6	T_pancreas_4	N_prostate_6	T_lung_4	T_prostate_1	T_uterus_1	T_mesothelioma_1	T_prostate_6	T_mesothelioma_1	T_kidney_1	T_colon_9	N_kidney_2	T_pancreas_5	N_breast_3	T_uterus_3	N_pancreas_1	T_pancreas_4
3	T_melanoma_2	T_uterus_9	N_breast_2	T_uterus_10	N_bladder_1	N_colon_5	T_kidney_5	T_pancreas_4	N_prostate_4	T_bladder_5	T_ovary_3	T_uterus_10	N_colon_3	T_pancreas_4	T_colon_5	T_pancreas_4	N_prostate_2	T_pancreas_6	T_ovary_1
4	N_colon_4	T_uterus_1	T_breast_4	T_kidney_5	N_prostate_7	T_mesothelioma_1	N_lung_3	N_uterus_8	T_pancreas_7	T_melanoma_1	T_mesothelioma_4	T_lung_4	T_colon_5	N_breast_2	T_mesothelioma_1	T_prostate_3	T_pancreas_7	T_pancreas_2	T_pancreas_6
5	T_uterus_8	N_prostate_8	T_prostate_4	N_colon_1	N_prostate_8	T_prostate_6	N_colon_3	T_pancreas_9	T_mesothelioma_6	T_pancreas_2	T_uterus_4	T_colon_9	T_colon_7	N_prostate_4	T_colon_10	T_pancreas_9	T_prostate_6	T_ovary_3	T_bladder_3
6			T_prostate_1			N_prostate_6	N_breast_2		T_prostate_3			N_colon_3						T_pancreas_9	
7							N_uterus_8		N_colon_1										
8									T_pancreas_2										

## Results

**Figure 4:**
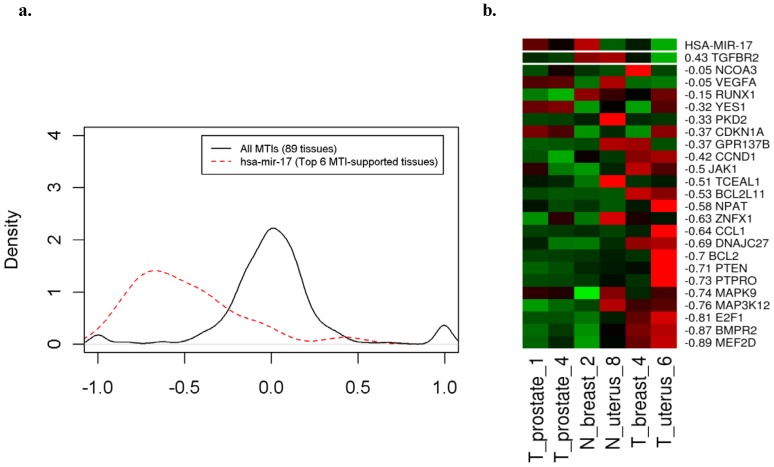
The correlation density plot and heatmap for experimentally validated MTIs (miRTarBase). a. Comparison of correlation densities for all 743 experimentally validated MTIs (solid line) and hsa-miR-17 with 24 targets across the top 6 MTI-supported tissues (dashed line). b. The expression profiles of hsa-miR-17 and 24 targets across the top 6 MTI-supported tissues. The correlations between the expression profile of hsa-miR-17 and the profiles of target genes were annotated next to the gene symbol. Green represented low expression and red represented high expression in corresponding genes and tissues.

Using the algorithm, we obtain MTI-supported tissues for 19 miRNAs that are listed in Table 2. In the following, we use hsa-miR-17 as an example to describe the analysis result. Figure 4 presents the plots for the density function of correlation and the heatmap of hsa-miR-17. Figure 4(a) shows the density plot of correlations (solid line) of all 743 experimentally validated MTIs across all 89 tissues and the density plot of correlations (dashed line) between hsa-miR-17 and its targets across the top 6 MTI-supported tissues. The solid line is symmetric and centralized to zero; however, the dashed line is right-skewed. Apparently, the right-skewed density plot of the correlations between hsa-miR-17 and its targets across the top 6 MTI-supported tissues shows that most correlations are negative, which is in accordance with the degradation behavior between an miRNA and its targets. In contrast, the fact that the correlations of all 743 experimentally validated MTIs across all 89 tissues are near zero indicates that the down-regulation behavior of an miRNA with its targets only displays across the MTI-supported tissues.

Figure 4(b) is an example that shows that the expression profiles of most hsa-miR-17 target genes are negatively correlated with the expression profile of hsa-miR-17. In Figure 4(b), the expression profiles of hsa-miR-17 and 24 target genes across the top 6 MTI-supported tissues are presented. The correlation between the expression profiles of hsa-miR-17 and each target gene is annotated next to the gene symbol in Figure 4(b). Most of expression profiles of hsa-miR-17 target genes are negatively correlated with the expression profile of hsa-miR-17. The expression profiles of the hsa-miR-17 target gene TGFBR2 is positively correlated with the miRNA expression profile. Although it has been experimentally validated that these target genes could be inhibited by hsa-miR-17, TGFBR2 is not. The reason for the positive correlation between hsa-miR-17 and these genes could be that these genes are regulated by other stronger regulatory factors. To support our suspicion, we re-examine the studies of hsa-miR-17 regulation on TGFBR2, which shows that TGFBR2 expression could not be detected because of microsatellite-instability and mutations [32]–[34].

In addition, the correlation density plots of other 18 miRNAs and their targets (At http://tsmti.mbc.nctu.edu.tw) reveal similar results to that of hsa-miR-17. For example, the proposed algorithm searches out 5 top specific tissues for hsa-miR-21, which has the largest target number (43 targets) among the miRNAs that have been considered in this study. In addition, we also find the top 8 MTI-supported tissues for hsa-let-7a and its targets. Both of the correlation density plots across MTI-supported tissues are right-skewed and are greater than those plots across all 89 tissues. In addition to the correlation density results, we observe that the selected tissue number depends on the miRNA. In the Methods section, we show that the optimal tissue number, which is derived by the algorithm, is dependent on the miRNAs. Due to the limited space, we only provide the elaboration on the example miR-17 in details. For other miRNAs, we calculate average correlation between an miRNA and its targets across all 89 tissues and average correlation between an miRNA and its targets across selected MTI-supported tissues. Then we use the value of the second average correlation minus the first average correlation as a metric to quantify the quality improvement. The results are presented in Table 2. The values are range from −0.32 to −0.68. The value for miR-17 is −0.536. It reveals that average correlation between an miRNA and its targets across selected MTI-supported tissues are more strongly negative correlated than the average correlation between an miRNA and its targets across all 89 tissues.

**Figure 5:**
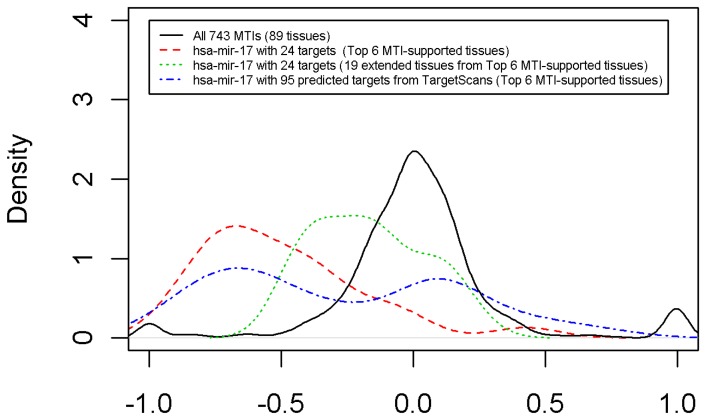
The correlation density plot. Comparison of correlation densities for all 743 experimentally validated MTIs across all 89 tissues (black solid line), hsa-miR-17 with 24 targets across the top 6 MTI-supported tissues (red dashed line), hsa-miR-17 with 24 targets across 19 extended tissues (green dashed line) and hsa-miR-17 with 95 predicted targets across the top 6 MTI-supported tissues (blue dashed line).

Because the top 6 MTI-supported tissues that have been selected for hsa-miR-17 include tumor tissues, which may be queried with extreme expression levels, we extend the top 6 MTI-supported tissues to other tissues with the same organ as the top 6 MTI-supported tissues. The top 6 MTI-supported tissues for hsa-miR-17 include tumor tissues and normal tissues. Tumor tissues are composed of prostate, breast and uterus tissues. Normal tissues are composed of breast and uterus tissues. The number of tissues is extended to 24 because the total number of tumor prostate tissues, tumor/normal breast tissues and tumor/normal uterus tissues is 24. Here, we again examine the expression levels of 24 tissues of hsa-mir-17. Only 19 expression levels are larger than 7.25. A tissue is eliminated if its miRNA expression level that corresponds to the tissue is less than 7.25, which is the cutoff point that was used in Huang et al. [7]. Figure 5 shows the correlation density plot (green dashed line) for the extended results. Comparing Figure 4(a) with Figure 5, we add a green dashed line in Figure 5, which is the correlation density plot of hsa-miR-17 and its targets across the 19 extended tissues. The green dashed line is always between the black solid line and the red dashed line. The result clearly reveals that the analysis that is based on 6 MTI-supported tissues leads to the best result, followed by the extended tissues, which are better than the analysis that is based on 89 tissues.

Furthermore, we also investigate the target prediction that is based on the selected tissues. By searching for the conserved 8mer and 7mer sites that match the seed region of each miRNA from the TargetScanS prediction tool [3]–[5], we have 95 predicted targets of hsa-miR-17 from our dataset, which are based on the top 6 MTI-supported tissues. The blue dashed line in Figure 5 is the correlation density of hsa-miR-17 and 95 predicted targets across the top 6 MTI-supported tissues. Although a small portion of correlations are positive correlations, most of the correlations are more negative than that across all 89 tissues. Nevertheless, the correlation density of hsa-miR-17 and 95 predicted targets across the top 6 MTI-supported tissues (blue dashed line) does not have a better performance than that obtained using 24 experimentally validated MTIs across the top 6 MTI-supported tissues (red dashed line) and that obtained using 24 experimentally validated MTIs across 19 extended tissues (green dashed line). Adopting 95 predicted targets of hsa-miR-17 across the top 6 MTI-supported tissues can still improve the correlations that are not near zero. By the extended case and the predicted case, it is concluded that the proposed algorithm is reliable for selecting MTI-supported tissues.

## Discussion

Our approach is focused on discovering MTI-supported tissues for an miRNA based on experimentally validated target genes. However, we find some mRNAs are not down-regulated by their experimentally validated miRNA. Several potential reasons are described. First, mRNA expression can be regulated by multiple factors including DNA copy number, transcriptional regulation and post-transcriptional regulation. Since our approach selects a group of different tissues, miRNA repression ability could be smaller than other regulation mechanisms in part of selected tissues. Second, some miRNAs not only down-regulate their target genes, but also up-regulate their target genes [35]. miRTarBase does not include any information about an miRNA up-regulates or down-regulates its target genes. It is supposed that the miRNA in miRTarBase can only down -regulate its target genes. Nevertheless, many miRNAs have been reported that miRNAs can up-regulate their target genes [35]–[38]. For example, the record of MIRT004506 in miRTarBase is that miR-466l up-regulates IL-10 via binding AU-rich region in 3′UTR [36]. Therefore, some up-regulation phenomenons are discovered from the correlation density plots across the MTI-supported tissues.

From the correlation density plots across the MTI-supported tissues in Table 2, we find that several experimentally validated MTIs are positively correlated with the miRNA expression profiles. First, miR-145 is positively correlated to the target gene IRS1 with a correlation of 0.55. A previous report shows that miR-145 can down-regulate the protein level of IRS1 but cannot down-regulate the mRNA of IRS1 [39]. Therefore, the negative correlation between miR-145 and IRS1 is not observed. Furthermore, some cell lines, such as BCT-20, do not express IRS1. Thus, the down-regulation of IRS1 by miR-145 cannot be observed [40]. The second non-negatively correlated MTI is miR-1 and SERP1. The study, which does examine the interaction between miR-1 and SERP1, shows that the down-regulation of SERP1 by miR-1 is not significant [41]. The miRTarBase database might record many experimentally validated MTIs whose target genes are significantly down-regulated by the corresponding miRNAs. It is difficult to determine the reason for some positively correlated experimentally validated MTIs, such as the experimentally validated MTI between miR-1 and FOXP1. More advanced experiments are required to explore these MTIs.

**Figure 6:**
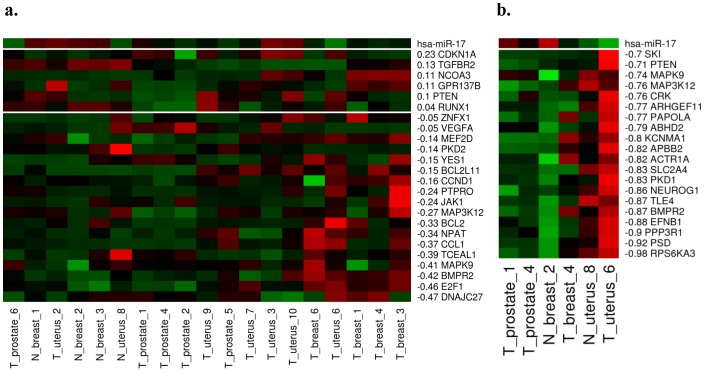
The heatmaps of hsa-miR-17 expression and regulated genes. a. The expression profiles of hsa-miR-17 and 24 targets across 19 extended tissues. The correlations between the expression profiles of hsa-miR-17 and target genes were annotated next to the gene symbols. Green represented low expression and red represented high expression in corresponding genes and tissues. b. Part of the expression profile of hsa-miR-17 and experimentally validated and predicted target genes. There are 20 negatively correlated target genes; 16 of these target genes are predicted to be hsa-miR-17 target genes. PTEN, MAPK9, MAP3K12 and BMPR2 are experimentally confirmed target genes.

Figure 6 (a) is an extended illustration of Figure 4. We collect expression profiles, which are sampled from the same organs that are listed in Figure 4. We observe more conflicted correlations in experimentally validated MTIs. First, the correlation between the expression of CDKN1A and hsa-miR-17 is 0.23, and the correlation between the expression of RUNX1 and hsa-miR-17 is 0.04. Previous studies have revealed that CDKN1A and RUNX1 are regulated by multiple miRNAs [42], [43]. Due to our approach, which only observes one miRNA and its corresponding target genes, the expression of CDKN1A and RUNX1 could not simply negatively correlate to the expression of hsa-miR-17. Two conflicted studies show that hsa-miR-17 can or cannot inhibit the translation of PTEN. Olive et al. (2009) concluded that PTEN could be repressed by miR-19, but not by hsa-miR-17, in B-cell lymphoma [44]. The experiment that was reported by Trompeter et al. (2007) shows that PTEN is significantly repressed by hsa-miR-17 in HEK293T cells and kidney cells [45]. NCOA3 (AIB1) is not correlated with the expression of hsa-miR-17. The expression correlations are negative in breast tissues and are in agreement with the study that demonstrated that NCOA3 is down-regulated by hsa-miR-17 [46]. However, these interactions are not negatively correlated with the rest of the tissues. This phenomenon indicates that an miRNA-target interaction is MTI-supported tissue-specific and reveals that our approach might include tissues whose experimentally validated MTIs are not functional. Because the seed region of miR-17, miR-106a and miR-20a are identical, the expression correlation of hsa-miR-17 and its target genes might be affected by other miRNAs. Thus, our approach can determine which target genes are dominantly regulated by hsa-miR-17 and find the non-MTI-supported tissues.

Currently, many studies use differentially expressed genes to reduce false-positive miRNA target sites [22], [25], [47]. We also apply our approach to the predicted miRNA target genes. In Figure 6 (b), 16 predicted target genes have a highly negative correlation with the expression of the miRNA.

In addition, we present the selected tissues of miRNAs and their targets in Table 4. Table 4 shows that three of five MTI-supported tissues of hsa-miR-34a are prostate tissues; four of five MTI-supported tissues of hsa-miR-106a are colon tissues; three of five MTI-supported tissues of hsa-miR-222 are uterus tissues; three of five MTI-supported tissues of hsa-miR-26a are pancreatic tissues; and four of six MTI-supported tissues of hsa-miR-141 are pancreatic tissues. These tissues account for a high proportion of selected MTI-supported tissues. Therefore, we conclude that each miRNA can down-regulate its experimentally validated target genes in the selected tissues. Although not all of selected tissues account for a high proportion of MTI-supported tissues, the correlations between an miRNA and its targets across selected tissues are highly negative. The individual selected tissues, which do not account for a high proportion of MTI-supported tissues, can also be suggested as potential MTI-supported tissues of the miRNA and its targets.

**Table 4:** The list of the selected MTI-supported tissues corresponding to 19 miRNAs with equal to or more than 10 targets among 743 experimentally validated MTIs.

miRNA	the number ofselectedMTI-supportedtissues	Colon	Uterus	Prostate	Pancreatic	Ovary	Lung	Breast	Bladder	Mesothelioma	melanoma	Kidney
**hsa-miR-21**	5	1	1			1					1	1
**hsa-miR-1**	5		2	2						1		
**hsa-miR-17**	6		2	2				2				
**hsa-miR-145**	5	1	2		1							1
**hsa-miR-34a**	5			3				1	1			
**hsa-miR-125b**	6	1		1			1	2		1		
**hsa-miR-16**	7	1	1	1			1	2				1
**hsa-miR-221**	5		2		2	1						
**hsa-let-7a**	8	1		2	2				1	2		
**hsa-miR-29b**	5			1	1	1			1		1	
**hsa-miR-210**	5	1	1			1				2		
**hsa-miR-20a**	6	2	1				1		1			1
**hsa-miR-106a**	5	4	1									
**hsa-miR-29c**	5			1	1			1		1		1
**hsa-miR-222**	5		3		1					1		
**hsa-miR-26a**	5			1	3			1				
**hsa-miR-24**	5		1	2	1						1	
**hsa-miR-15a**	6				4	2						
**hsa-miR-141**	5				3	1			1			

Because the correlation results are not significant for explaining the down-regulation between an miRNA and its targets, we are highly interested in these results, particularly because the results conflict with our expectation. The results of our proposed algorithm support that experimentally validated MTIs are only functional in MTI-supported tissues. In addition, we also provide two methods to verify the tissue-specificity; the results uphold our proposed algorithm as an efficient approach to select valid MTI-supported tissues of an miRNA. Our approach uses publicly available microarray data to select the predicted target genes, which are highly negatively correlated with the miRNA expression profiles. The benefit of our approach is that the experimental cost could be reduced; however, the drawback is that the expression profiles of the experimental target might differ from our collected expression profiles. This drawback can be improved by increasing the expression profiles from all type of tissues. Once the numbers of miRNA and mRNA expression profiles are increased, our approach might provide better performance for selecting potential experimentally validated MTIs from the predicted MTIs, and the MTI validation could be accelerated by our analysis.

## Supporting Information

File S1 **Figure S1–S3.** Figure S1. Summary of the correlations of 743 MTIs (miRTarBase) across all 89 tissues. a. The proportion of negative correlations is near 50%. b. The amount of correlations between −0.5 and 0.5 is larger than 90%. Both results reveal that there are no significant correlations among MTIs using this data source across all 89 tissues. Figure S2. The expression levels of hsa-miR-122 and hsa-let-7a in 89 tissues The red line is a cutoff line, and the cutoff value is 7.25 (after log_2_ transformed). The data were eliminated when the expression level was lower than the cutoff line (Huang et al. 2007). Figure S3. Comparison of selected results and permutation results. (a)(b) Loss function values of each miRNA and its targets across MTI-supported tissues and permutation tissues.
(RAR)
