# Correction: Investigating microRNA-Target Interaction-Supported Tissues in Human Cancer Tissues Based on miRNA and Target Gene Expression Profiling

**DOI:** 10.1371/journal.pone.0102769

**Published:** 2014-07-09

**Authors:** The PLOS ONE Staff

There is an error in the “Authorship Contributions.” Both Dr. Wan J. Hsieh and Dr. Feng-Mao Lin are joint first authors for this article.
